# Higher oxidative balance score is associated with lower female infertility: a cross-sectional study

**DOI:** 10.3389/fnut.2024.1484756

**Published:** 2024-12-04

**Authors:** Xiong Lei, Xiling Liu, Chunchun Yu, Lijing Xia, Liwen Zhou, Can Yao, Zhixiao Xu

**Affiliations:** ^1^Department of Emergency, The First Affiliated Hospital of Wenzhou Medical University, Wenzhou, China; ^2^Department of Pulmonary and Critical Care Medicine, The First Affiliated Hospital of Wenzhou Medical University, Wenzhou, China; ^3^Department of Gastroenterology, Minhang District Central Hospital of Shanghai, Fudan University, Shanghai, China

**Keywords:** female infertility, oxidative balance score, NHANES, dietary, lifestyle

## Abstract

**Background:**

An imbalance of the pro-oxidant and antioxidant profiles in the body contributes to the development of diseases, including female infertility.

**Methods:**

In this study, we performed a secondary analysis of the National Health and Nutrition Examination Survey and calculated the values of oxidative balance score (OBS). A weighted logistic regression model was used to explore the potential relationship between OBS (continuous factor and quartile set) and female infertility, and the P for trend was calculated. Subgroup analyses were also carried out further to explore the association between OBS and female infertility, and the P for interactions were obtained.

**Results:**

There were 1,626 females (aged 18 to 45) included in the study, including 198 with infertility and a prevalence of approximately 13.28%. Multifactorial logistic regression showed a 5% decrease in infertility for each unit increased in OBS (OR, 0.95; 95% CI, 0.92 to 0.98). When OBS was used as a categorical variable, female infertility decreased by 60% in the highest OBS group compared with the lowest OBS group (OR, 0.40; 95% CI, 0.21 to 0.74). In addition, subgroup analyses showed that the negative association between OBS and infertility was significant in the non-chronic disease (hypertension and diabetes) population, those with less than a high school educational background, poverty-to-income ratio (1 to 3), and those with more than 30 years of age.

**Conclusion:**

Higher OBS was negatively associated with female infertility. Further prospective studies are needed to determine causality and to provide new perspectives on female infertility in the context of diet and lifestyle.

## Introduction

Infertility is in the spotlight once again with the global decline in fertility (1). It is a public health problem, the odds of infertility are trending upward to 8.1% from 2017 to 2019 in the United States (2). Although assisted reproductive technologies and advanced medications continue to develop (3, 4), they cannot address the root cause of the problem. Reproductive health should be emphasized in life and included in daily health management. In addition, there are many secondary conditions caused by infertility, such as depression (5). Many factors cause infertility, external factors include heavy metal pollution and air pollution (6, 7), and internal factors such as inflammation levels, oxidative and antioxidant imbalances (8, 9). Infertile females not only lead to marital crises but also suffer more (10). Therefore, it is important to prevent female infertility based on so many adverse effects.

The properties of diet and drugs are very closely associated with female infertility. Toxic mediators in the environment are reproductively toxic and also increase the risk of infertility by altering oxidation levels in the body (11). Therefore, some drugs are used to combat oxidative stress to improve pregnancy outcomes in *in vitro* fertilization (12). Recently, astaxanthin, as an antioxidant, helps reduce levels of oxidative stress in infertile females with polycystic ovary syndrome (13). In addition, some natural antioxidant supplementation (vitamins and flavonoids) improves female fertility (14). In addition to diet and medication, lifestyle has an irreplaceable role to play in female infertility. Both smoking and physical activity potentially affect female fertility (15, 16). However, these studies only focused on the effect of a single oxidative factor on female infertility. Regulating the level of oxidative stress in the body may offer new promise for female infertility. For example, physiologically, polycystic ovary syndrome leads to increased levels of oxidative stress thus leading to infertility (17). The concept of a relationship between overall oxidation levels and female infertility has not been proposed.

Oxidative balance score (OBS) as an overall pro-oxidant and antioxidant properties level is a highly important measure, higher OBS means greater antioxidant capacity of the body. It has steadily developed into an epidemiological indicator over the years. It also has been shown to correlate with various health problems such as sleep and lung disease (18, 19). Given the inextricable relationship between diet/lifestyle properties and female infertility, there is lacking comprehensive research on the unique role of antioxidant levels in female infertility. Therefore, the potential research between overall levels of oxidative and antioxidant properties and female infertility is enormous and promising. We hypothesized that there was a negative relationship between OBS and the risk of female infertility. Weighted logistic regression was used to explore the potential relationship between OBS (continuous factor and quartile set) and female infertility. By exploring the relationship between infertility and OBS, it may provide new ideas for infertility prevention.

## Materials and methods

### Population selection

We performed a secondary analysis of the National Health and Nutrition Examination Survey (NHANES) data. NHANES is a health prevention program that covers a wide range of people and has laboratory tests, questionnaires, and other information that can be used to investigate risk factors for disease and provide prevention strategies to prevent disease.

**Figure 1:**
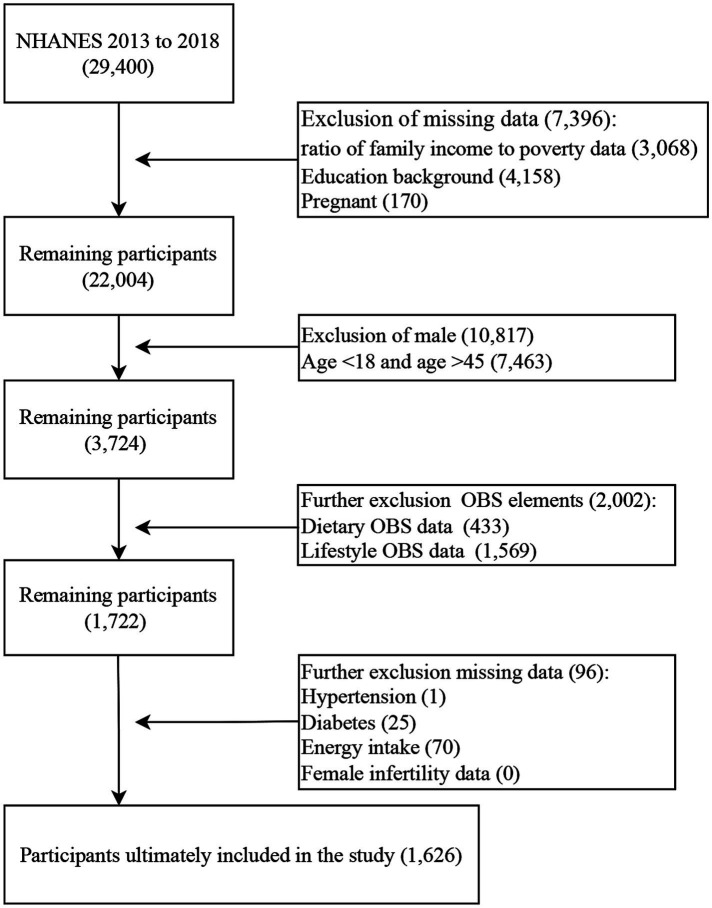
Flowchart for selecting participants.

A total of 29,400 participants were in NHANES from 2013 to 2018 (three survey cycles). First, we excluded missing poverty-to-income ratio (PIR) data (3,068), education background data (4,158), and pregnancy (170). Second, we exclude males (10,817) and females aged under 18 or age older than 45 (7,463). Then, participants missing OBS elements data were excluded (2,002). Next, we further excluded missing data on hypertension, diabetes, energy intake, and female infertility data (96). Finally, the sample included in the study was 1,626 participants (Figure 1).

### Exposure definition

OBS calculations are constantly being innovated. Based on the most recent studies of OBS calculations, the study incorporated 5 pro-oxidants and 15 antioxidants. Five pro-oxidant components included total fat, iron, serum cotinine, alcohol consumption, and body mass index (BMI). Fifteen antioxidants included dietary fiber, carotene, riboflavin, niacin, vitamin B6, total folate, vitamin B1, vitamin C, vitamin E, calcium, magnesium, zinc, copper, selenium, and physical activity. Higher OBS means higher antioxidant levels. Dietary OBS information was obtained from participants. To ensure the accuracy of dietary intake, participants underwent two 24-h dietary recall interviews. The first dietary recall interview was collected in person at a mobile screening center, and the second interview was collected by telephone 3 to 10 days later. The final intake obtained was the average of the two interviews. Alcohol consumption, serum cotinine, BMI, and physical activities were used to define lifestyle OBS. Alcohol consumption was defined as alcoholic beverages on average in the past 12 months. Serum cotinine levels were used as a measure of smoking. The physical activity definition was referred to in this study (20) and the formula was as follows: metabolic equivalent score * frequency of each physical activity per week * duration of each physical activity.

In the female population, each OBS component was divided according to tertiles. Antioxidant components were assigned a score of 2, 1, and 0 from high to low, while pro-oxidant components were assigned a score of 0, 1, and 2. The total OBS was the sum of the antioxidant and pro-oxidant components (Table 1). The maximum score could reach 40. Furthermore, the total OBS was divided into dietary OBS and lifestyle OBS.

**Table 1:** Composition of oxidative balance score in females.

OBS components	Property	Female		
		0	1	2
Dietary OBS components				
Dietary fiber (g/d)	A	<10.50	10.50–17.30	≥17.30
Carotene (RE/d)	A	<490.00	490.00–1,826.00	≥1,826.00
Riboflavin (mg/d)	A	<1.41	1.41–2.10	≥2.10
Niacin (mg/d)	A	<17.31	17.31–25.20	≥25.20
Vitamin B6 (mg/d)	A	<1.32	1.32–2.04	≥2.04
Total folate (mcg/d)	A	<244.00	244.00–375.00	≥375.00
Vitamin B12 (mcg/d)	A	<2.29	2.29–4.31	≥4.31
Vitamin C (mg/d)	A	<28.80	28.80–82.70	≥82.70
Vitamin E (ATE) (mg/d)	A	<5.64	5.64–9.37	≥9.37
Calcium (mg/d)	A	<638.00	638.00–982.00	≥982.00
Magnesium (mg/d)	A	<215.00	215.00–299.00	≥299.00
Zinc (mg/d)	A	<7.25	7.25–10.63	≥10.63
Copper (mg/d)	A	<0.85	0.85–1.24	≥1.24
Selenium (mcg/d)	A	<76.35	76.35–112.50	≥112.50
Total fat (g/d)	P	≥58.16	58.16–84.74	<84.74
Iron (mg/d)	P	≥8.82	8.82–12.07	<12.07
Lifestyle OBS components				
Physical activity (MET-minute/week)	A	<1,440.00	1,440.00–4,580.00	≥4,580.00
Alcohol (g/d)	P	≥3.00	3.00–2.00	<2.00
Body mass index (kg/m2)	P	≥31.30	23.60–31.30	<23.60
Cotinine (ng/mL)	P	≥0.17	0.02–0.17	<0.02

A, antioxidant; ATE, alpha-tocopherol equivalent; MET, metabolic equivalent; NHANES, National Health and Nutrition Examination Survey; OBS, Oxidative balance score; P, pro-oxidant; RE, retinal equivalent.

### Outcome definitions

Female infertility as the outcome of the study was derived from the Reproductive Health Questionnaire. The female population aged 18 to 45 years was selected for this study. Participants were asked “Have you ever attempted to become pregnant over a period of at least a year without becoming pregnant?” and “Have you ever been to a doctor or other medical provider because you have been unable to become pregnant?,” female infertility was defined when any of them answered “Yes.”

### Covariates definitions

First, age, race/ethnicities (Non-Hispanic White, Non-Hispanic Black, other Hispanic, Mexican-American, and other races/ethnicities), educational background (below high school, high school, and above high school), and PIR as demographic factors were included. Second, uncontrolled hypertension is associated with female infertility (21). The molecular mechanisms by which diabetes affects female infertility have been revealed (22). Therefore, hypertension and diabetes were included as covariates. Hypertension was defined using the following questionnaire: “Have you ever been told by a doctor or other health professional that you had hypertension, also called high blood pressure?,” and diabetes was defined by the questionnaire: “Have you ever been told by a doctor or health professional that you have diabetes or sugar diabetes?.” Concerning previous studies on the association of OBS with disease, total energy intake should be included as a covariate and extreme energy intake needs to be excluded (<500 or >3,500 kcal day^−1^ for females) (23, 24). Energy intake was 24 h before the interview and was averaged from two 24-h dietary recall interviews (days 1 and 3 to 10 days later).

### Statistical analysis

We downloaded the three survey cycle data (2013 to 2018) from the NHANES website. A normality test was performed on the continuous variables, we expressed the non-normal continuous variables in terms of the median and the categorical variables expressed as unweighted frequencies (weighted percentages).

The Rao–Scott chi-squared test and the Kruskal–Wallis test were used for categorical and non-normal continuous variables, respectively, to correctly test for the baseline characteristics of the OBS quartiles. The weighted logistic regression was used to explore the relationship between OBS and female infertility. The model 1 was not adjusted. The model 2 was adjusted by age and race/ethnicity. The model 3 was adjusted all the covariates.

In addition, the study also investigated the association between dietary OBS and lifestyle OBS and female infertility. Subgroup analyses were also performed by age, educational background, PIR, hypertension, and diabetes. To test the stability of the model, sensitivity analyses were conducted by excluding the OBS components one by one.

R software (4.2.2) was used to perform the analyses. The statistical significance threshold was set at 0.05.

## Results

### Baseline characteristics of participants by OBS’ quartiles

A total of 1,626 female participants who met the inclusion criteria made up the final sample. The female infertility rate was approximately 13.28%. Non-Hispanic White individuals (62.04%) make up the majority of them and 56% of participants had above high school educational background. The rate of female infertility among participants was 13.28% (Table 2).

**Table 2:** Baseline characteristics of participants by OBS’ quartiles.

		Q1 (<15)	Q2 (15–20)	Q3 (21–25)	Q4 (>25)	*p*-value
	*N* = 1,626	*N* = 455	*N* = 413	*N* = 377	*N* = 381	
Age (year)	31 [24; 38]	30 [24; 37]	30 [23; 38]	31 [24; 38]	33 [27; 39]	0.008
**Race/Ethnicity**						0.061
Mexican American	253 (10.36%)	59 (9.68%)	60 (10.15%)	57 (8.87%)	77 (12.66%)	
Other Hispanic	139 (6.16%)	33 (5.89%)	37 (7.35%)	32 (5.22%)	37 (6.18%)	
Non-Hispanic White	649 (62.04%)	173 (58.23%)	167 (59.78%)	154 (65.56%)	155 (64.44%)	
Non-Hispanic Black	320 (11.36%)	121 (16.33%)	89 (12.98%)	65 (9.69%)	45 (6.65%)	
Other race	265 (10.08%)	69 (9.87%)	60 (9.74%)	69 (10.66%)	67 (10.06%)	
**Educational background**						<0.001
<Highschool	174 (7.59%)	65 (12.67%)	49 (8.88%)	27 (3.60%)	33 (5.35%)	
>Highschool	1,130 (74.30%)	278 (61.47%)	274 (70.99%)	273 (78.23%)	305 (85.93%)	
Highschool	322 (18.11%)	112 (25.86%)	90 (20.13%)	77 (18.17%)	43 (8.72%)	
**Hypertension**						0.462
Yes	222 (11.18%)	74 (13.61%)	60 (10.82%)	50 (11.10%)	38 (9.28%)	
No	1,404 (88.82%)	381 (86.39%)	353 (89.18%)	327 (88.90%)	343 (90.72%)	
**Diabetes**						0.327
Yes	67 (3.43%)	24 (4.67%)	26 (4.49%)	9 (2.29%)	8 (2.35%)	
No	1,559 (96.57%)	431 (95.33%)	387 (95.51%)	368 (97.71%)	373 (97.65%)	
Energy intake	1,811 [1,419; 2,226]	1,330 [1,031; 1,692]	1,736 [1,410; 2,067]	1,900 [1,589; 2,339]	2,246 [1,847; 2,674]	<0.001
**Poverty-to-income ratio**						<0.001
<1	351 (16.23%)	130 (21.46%)	104 (20.91%)	60 (11.74%)	57 (11.04%)	
1–3	667 (36.91%)	204 (44.57%)	154 (32.57%)	163 (37.70%)	146 (33.02%)	
>3	608 (46.86%)	121 (33.97%)	155 (46.53%)	154 (50.56%)	178 (55.94%)	
**Female infertility**						0.490
Yes	198 (13.28%)	55 (14.74%)	55 (12.09%)	47 (14.97%)	41 (14.43%)	
No	1,428 (86.72%)	400 (85.26%)	358 (87.91%)	330 (85.03%)	340 (88.57%)	

NHANES, National Health and Nutrition Examination Survey. OBS, oxidative balance score; Q1, the lowest OBS group; Q2, the lower OBS group; Q3, the higher OBS group. Q4, the highest OBS group.

### Baseline characteristics of with or without female infertility

As shown in Table 3, participants with infertility had older age, diabetes, and lower OBS compared to no infertility participants (*p*-value less than 0.05).

**Table 3:** Baseline characteristics of participants with or without female infertility.

		No female infertility	Female infertility	*p-*value
	*N* = 1,626	*N* = 1,428	*N* = 198	
Age (year)	31 [25; 38]	30 [24; 38]	36 [29; 41]	<0.001
**Race/Ethnicity**				0.821
Mexican American	253 (10.36%)	219 (10.05%)	34 (12.42%)	
Other Hispanic	139 (6.16%)	129 (6.34%)	10 (4.97%)	
Non-Hispanic White	649 (62.04%)	566 (62.14%)	83 (61.39%)	
Non-Hispanic Black	320 (11.36%)	282 (11.49%)	38 (10.47%)	
Other race	265 (10.08%)	232 (9.98%)	33 (10.74%)	
**Educational background**				0.441
<High school	174 (7.59%)	160 (7.95%)	14 (5.26%)	
>High school	1,130 (74.30%)	986 (73.85%)	144 (77.24%)	
High school/General educational development	322 (18.11%)	282 (18.20%)	40 (17.50%)	
**Hypertension**				0.159
Yes	222 (11.18%)	186 (10.53%)	36 (15.30%)	
No	1,404 (88.82%)	1,242 (89.47%)	162 (84.60%)	
**Diabetes**				0.001
Yes	67 (3.43%)	51 (2.62%)	16 (8.75%)	
No	1,559 (96.57%)	1,377 (97.38%)	182 (91.25%)	
**Poverty-to-income ratio**				0.307
<1	351 (16.23%)	318 (16.90%)	33 (11.82%)	
1–3	667 (36.91%)	593 (36.77%)	74 (37.79%)	
<3	608 (48.86%)	517 (46.33%)	91 (50.38%)	
Oxidative balance score	21 [15; 26]	21 [15; 26]	21 [14; 25]	<0.001

### Association between OBS and female infertility

As shown in Table 4, OBS was a significantly negative association with female fertility after adjusting all covariates in model 3 (OR, 0.95; 95%CI, 0.92 to 0.98). In addition, female infertility was decreased by 60% from the lowest OBS group to the highest OBS group (OR, 0.40; 95%CI, 0.921 to 0.74).

**Table 4:** Association between OBS and female infertility.

Exposure	Female infertility, OR (95% CI)					
	Model 1	*p*-value	Model 2	*p*-value	Model 3	*p*-value
OBS (Continue)	0.99 (0.96 to 1.02)	0.347	0.98 (0.95 to 1.01)	0.147	0.95 (0.92 to 0.98)	0.007
OBS quartiles						
Q1 (<15)	Reference		Reference		Reference	
Q2 (15–20)	0.80 (0.51 to 1.23)		0.76 (0.48 to 1.21)		0.61 (0.37 to 1.02)	
Q3 (21–25)	1.02 (0.61 to 1.70)		0.97 (0.57 to 1.64)		0.71 (0.42 to 1.19)	
Q4 (>25)	0.75 (0.45 to 1.23)		0.63 (0.36 to 1.09)		0.40 (0.21 to 0.74)	
*p* for trend		0.466		0.222		0.013

CI, confidence interval; NHANES, National Health and Nutrition Examination Survey; OR, odds ratio; OBS, oxidative balance score; Q1, the lowest OBS group; Q2, the lower OBS group; Q3, the higher OBS group. Q4, the highest OBS group. Model 1 was a crude model. Model 2 was adjusted for age and race/ethnicity. Model 3 was adjusted for age, race/ethnicity, educational background, poverty-to-income ratio, energy intake, hypertension, and diabetes.

### Association between the dietary OBS/lifestyle OBS and female infertility

As shown in Table 5, dietary OBS and lifestyle OBS were negatively associated with female infertility, and ORs (95%CI) were 0.96 (0.93 to 0.995) and 0.87 (0.76 to 0.999) in model 4, respectively.

**Table 5:** Association between the dietary /lifestyle OBS and female infertility.

	Model 1	*p*-value	Model 2	*p*-value	Model 3	*p*-value	Model 4	*p*-value
Female infertility, OR (95% CI)								
Dietary OBS	0.99 (0.96 to 1.02)	0.646	0.98 (0.95 to 1.02)	0.306	0.96 (0.92 to 0.99)	0.013	0.96 (0.93 to 0.995)	0.026
Lifestyle OBS	0.87 (0.76 to 0.98)	0.026	0.86 (0.77 to 0.97)	0.015	0.86 (0.75 to 0.98)	0.027	0.87 (0.76 to 0.999)	0.048

CI, confidence interval; NHANES, National Health and Nutrition Examination Survey; OR, odds ratio; OBS, oxidative balance score. The model 1 was a crude model. Model 2 was adjusted for age and race/ethnicity. Model 3 was adjusted for age, race/ethnicity, education level, poverty-to-income ratio, energy intake, hypertension, and diabetes. Model 4 was adjusted for dietary OBS or lifestyle OBS based on model 3 additionally.

### Subgroup analyses

**Figure 2:**
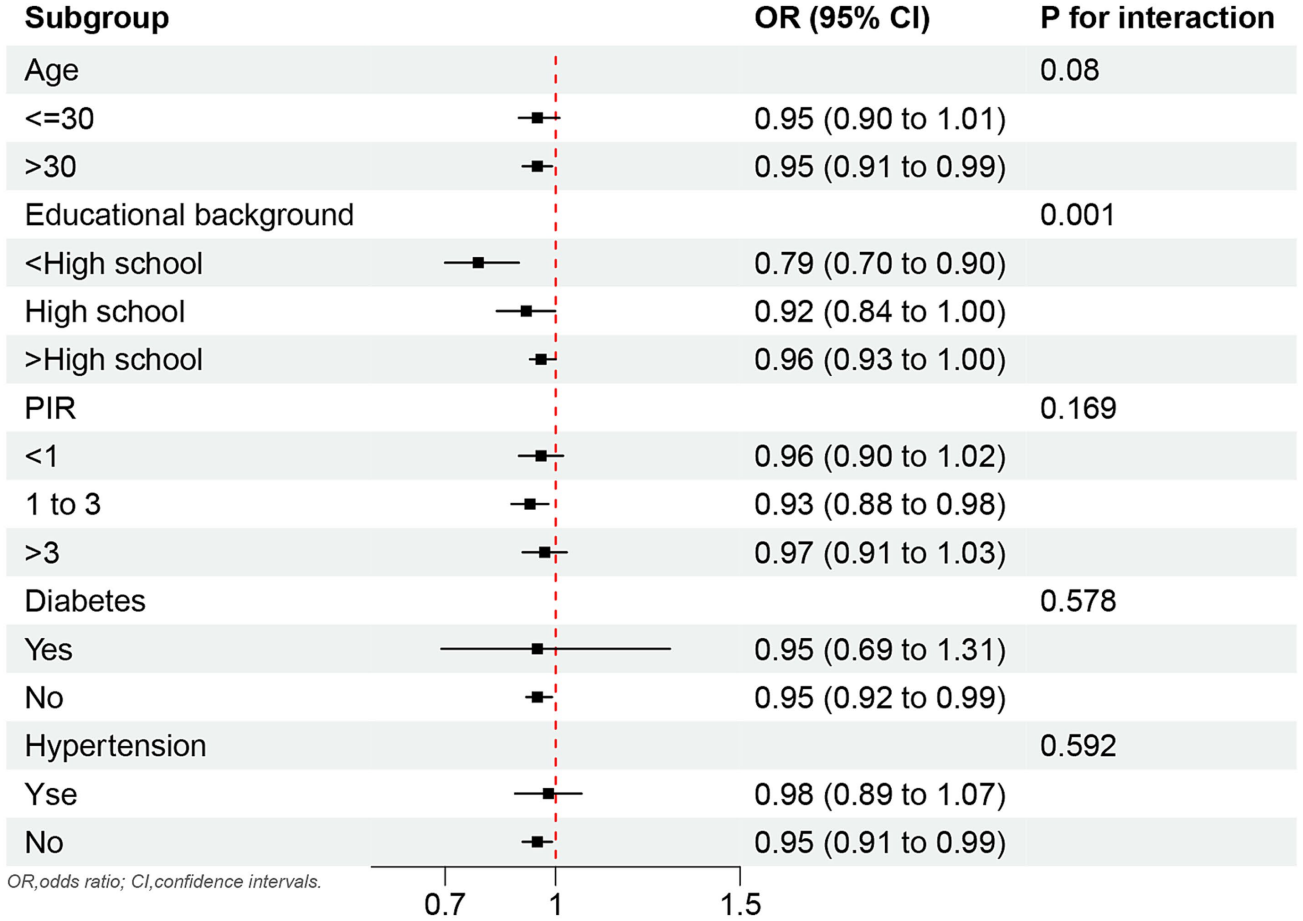
Subgroup analyses.

The negative correlation between OBS and female infertility was marked present in females older than 30 years (OR, 0.95; 95%CI, 0.91 to 0.99) and PIR in 1 to 3 (OR, 0.93; 95%CI, 0.88 to 0.98). In addition, this relationship also existed for those with less than a high school educational background and the *p* for interaction was 0.001. Furthermore, the negative correlation between OBS and female infertility also remained in females without chronic diseases (hypertension and diabetes; Figure 2).

In addition, with the removal of any individual OBS component, the relationship between OBS and female infertility was still robustly present (Supplementary S1).

## Discussion

Our study revealed a negative correlation between OBS and female infertility. As OBS increased, female infertility decreased, as evidenced by a 60% reduction in infertility in the highest OBS group compared to the lowest OBS group. In addition, dietary OBS and lifestyle OBS were both negatively associated with OBS. Subgroup analyses suggested that this relationship was significant in those aged older than 30 years and with a PIR of 1 to 2, but not in educational background above high school, hypertensive, and diabetic patients.

Increasing attention is being paid to the role of diet in infertility. Previous studies have shown that dietary fiber content is negatively associated with infertility in obese females (25). Polycystic ovary syndrome, a major factor in infertility, is associated with a lower intake of carotenoids, lutein, riboflavin, niacin, and fiber in this population (26). In addition, altering oxidative stress in the female reproductive tract through vitamin C supplementation decreases infertility in diabetic females (27). Vitamin C as an antioxidant also alleviates symptoms of endometriosis (28). The amount of zinc in the diet also leads to differences in the risk of infertility (29). However, some studies have also indicated that differences in iron in the blood of infertile patients may be the result of differences in iron absorption caused by various flora in the intestines (30). Copper and zinc levels were higher in infertile women with endometrial disease (31). Some studies have recommended the inclusion of screening for ferritin levels in unexplained infertility (32). However, previous studies have examined the relationship with infertility for one micronutrient and have not evaluated overall levels for a relatively large number of elements. Our study is based on the sum of antioxidant and pro-oxidant components in the body, which is more indicative of a relationship with infertility than the individual components.

Environmental exposure to tobacco smoke is an important contributor to infertility, especially as blood cotinine concentrations increase the risk of infertility (33). In addition, secondhand smoke exposure further reduces conception rates in women with polycystic ovary syndrome (PCOS) (34). However, it has also been shown that malondialdehyde and total antioxidant capacity are not associated with cotinine in infertile females (35). Physical activity has a lot of benefits, not only can it enhance the reproductive function of women with polycystic ovary syndrome to reduce infertility, but it can also alleviate women’s stress from social and psychological sources (36). Physical activity and infertility risk show a non-linearity and appropriate physical activity is beneficial for reproductive health (37). In males, diminishing oxidative stress through high-intensity exercise improved reproductive function (38). Obesity increases the risk of female infertility and miscarriages (39). A Korean survey showed that alcohol consumption can increase infertility and that the odds of infertility increased among those with a BMI ≥ 25 (40). However, it has also been shown that alcohol use does not correlate with follicular fluid-soluble receptors of advanced glycation end-products causing oxidative damage to ovarian follicles (41). In contrast, however, women with PCOS drank less alcohol than women without PCOS (42). In the U.S. population, obesity has led to increased rates of infertility, mostly due to anovulation (43). Obesity contributes to increased infertility through several mechanisms, and the accumulation of fat leads to systemic oxidative stress (44). Increased BMI is negatively correlated with follicle-stimulating hormone and luteinizing hormone (45). Obesity also has a significant impact on metabolism, nursing interventions for women of childbearing age at different times of pregnancy have also been proposed (46). In our results, lifestyle OBS was found to be negatively associated with infertility, suggesting that the level of infertility can be altered through lifestyle modification.

Similarly, social factors can have an impact on infertility. Some studies have shown a negative correlation between the level of education and the stress associated with female infertility (47). Second, women with fewer years of education and lower incomes in the United States have higher rates of infertility (2). Our study showed that the negative association of OBS with infertility was reflected in the population with PIR < 1. However, a study revealed PIR is positively associated with the risk of infertility (48). In recent decades, females have become more focused on advancing in their education as well as their careers, and there is a clear trend to delay fertility (49). In addition, as our results demonstrate, the relationship between OBS and female infertility was negative in the low-education population. The results reflected the importance of implementing infertility prevention measures in this population.

In addition, although the study did a lot of correlation analyses between OBS and female infertility, there were a few points that need to be noted. For example, we diagnose female infertility using a questionnaire form to understand a specific group. Questionnaires were often susceptible to personal perceptions, values, social expectations, and peer pressures. The consistency and reliability of the data were compromised. This was especially true when sensitive topics were involved. Although the questionnaire form of the survey could illustrate the science to a certain extent, having objective laboratory test results seemed to be able to study the problem more accurately. Therefore, scientific research through biomarkers as well as clinical assessment were necessary.

It was worth noting that OBS was an indicator that continues to improve as research progresses. The OBS in the study only represented 5 pro-oxidants and 15 antioxidant components in the human body. It was undeniable that the levels of chemical elements in the human body and the environment in which an individual lives were complex. The OBS for this study only represents the levels of oxidation and antioxidants calculated as best as possible from this database. As the study continues, the OBS will be improved and made representative. It has already been found that obesity-derived hormones have a moderating role in infertility (50). For example, an important possible reason for this was the specific role of adiponectin and visfatin in oxidative stress (51, 52). Therefore, including obesity-derived hormones in analyses may be a promising direction as obesity rates increase.

This study also had strengths and limitations. First, participants in the secondary analyses were taken from the representative NHANES database, and the results have broad applicability in the United States. Second, the study weighted the data to make the results more reliable and stable. Third, multivariate weighted regression models as well as sensitivity analysis were included in the study to investigate the potential relationship between OBS and female infertility further. However, the study also has limitations. First, the biggest limitation was the study was designed as a cohort study and could not conclude causality. Female infertility diagnosed using a questionnaire format was subjective. Participants might provide inaccurate information due to vague memories or emotional overtones. A proportion of participants might have cognitive biases, such as self-denial and overconfidence, which could affect the veracity of the questionnaire. However, the design of the questionnaire in NHANES was rigorous and every step was strictly controlled from the selection of the sample, interview setting and mode of administration, quality assurance and quality control, data processing, and editing. In the data analysis stage, we also weighted the data according to the official tutorial to try our best to reduce bias. However, prospective studies are necessary in the future.

## Conclusion

The study highlighted the importance of intervening in the degree of oxidation and antioxidation in bodies. It seemed that higher OBS was negatively associated with female infertility, especially among some key populations. The potential molecular mechanisms and temporal sequencing between OBS and female infertility need to be further verified. In the future, combining diet and lifestyle may deepen the understanding of female infertility, particularly early intervention, and health management for specific populations. Furthermore, the study provided public health policy recommendations for low fertility in some countries.

## Data Availability

The original contributions presented in the study are included in the article/Supplementary material, further inquiries can be directed to the corresponding authors.
