# Supplementary material for: Higher oxidative balance score is associated with lower female infertility: a cross-sectional study
Source: Front Nutr. 2024 Dec 4;11:1484756. doi: 10.3389/fnut.2024.1484756 (PMC11658776; doi:10.3389/fnut.2024.1484756)
Supplement: Supplementary file 1 [file Table_1.DOCX]

Supplementary Material

Higher oxidative balance score is associated with lower female infertility: A cross-sectional study

Xiong Lei^1^, Xiling Liu^2^, Chunchun Yu^2^, Lijing Xia^2^, Liwen Zhou^2^, Can Yao^3^*, Zhixiao Xu*^2^

*** Correspondence:**Corresponding Author: Can Yao and Zhixiao Xu
yaocan1230@163.com, xuzhixiao@wmu.edu.cn.

# Supplementary S1. Sensitivity analyses to assess the effects of individual Oxidative balance score components on the female infertility in NHANES 2013-2018 population.

| OBS | Female infertility | |  |
| --- | --- | --- | --- |
| OBS original model 3 | OR (95% CI) | P-value |  |
| OBS excluding dietary fiber | 0.95 (0.91 to 0.99) | 0.008 |  |
| OBS excluding carotene | 0.95 (0.91 to 0.99) | 0.008 |  |
| OBS excluding riboflavin | 0.95 (0.91 to 0.98) | 0.007 |  |
| OBS excluding niacin | 0.95 (0.91 to 0.99) | 0.008 |  |
| OBS excluding vitamin b6 | 0.95 (0.91 to 0.98) | 0.006 |  |
| OBS excluding total folate | 0.95 (0.91 to 0.99) | 0.009 |  |
| OBS excluding vitamin b12 | 0.95 (0.91 to 0.99) | 0.009 |  |
| OBS excluding vitamin c | 0.95 (0.91 to 0.98) | 0.007 |  |
| OBS excluding vitamin e | 0.95 (0.91 to 0.98) | 0.006 |  |
| OBS excluding calcium | 0.95 (0.91 to 0.98) | 0.006 |  |
| OBS excluding magnesium | 0.94 (0.91 to 0.98) | 0.005 |  |
| OBS excluding zinc | 0.95 (0.91 to 0.99) | 0.008 |  |
| OBS excluding copper | 0.95 (0.91 to 0.98) | 0.005 |  |
| OBS excluding selenium | 0.95 (0.91 to 0.98) | 0.006 |  |
| OBS excluding total fat | 0.95 (0.92 to 0.98) | 0.006 |  |
| OBS excluding iron | 0.95 (0.92 to 0.98) | 0.005 |  |
| OBS excluding physical activity | 0.95 (0.92 to 0.98) | 0.004 |  |
| OBS excluding alcohol | 0.95 (0.92 to 0.98) | 0.005 |  |
| OBS excluding body mass index | 0.96 (0.92 to 0.99) | 0.020 |  |
| OBS excluding cotinine | 0.95 (0.92 to 0.99) | 0.009 |  |
| Abbreviation: CI, confidence interval; NHANES, National Health and Nutrition Examination Survey; OR, odds ratio; OBS, oxidative balance score. The model 3 was adjusted for, age, race/ethnicity, education level, poverty-to-income ratio, energy intake, hypertension, and diabetes. | | |  |
|  |  |  |  |
|  |  |  |  |
|  |  |  |  |
|  |  |  |  |
